# Supplementary material for: Predation and fragmentation portrayed in the statistical structure of prey time series
Source: BMC Ecol. 2009 May 6;9:10. doi: 10.1186/1472-6785-9-10 (PMC2689204; doi:10.1186/1472-6785-9-10)
Supplement: Additional file 2 — Voles and related classes ODDox Documentation. ODDox documentation of the agent-based model (ALMaSS) applied by Hendrichsen et al. The documentation is started by activating main.html. [file 1472-6785-9-10-S2.zip › Vole_ODDox/globals_0x6c.html]

ALMaSS ODDox: Class Members

- Main Page
- Related Pages
- Classes
- Files

- File List
- File Members

- All
- Functions
- Variables
- Typedefs
- Enumerations
- Enumerator
- Defines

- \_
- a
- b
- c
- d
- f
- g
- h
- i
- l
- m
- n
- o
- p
- r
- s
- t
- u
- v
- w

Here is a list of all file members with links to the files they belong to:

### - l -

- l\_farm\_cattle\_veg\_reduce()
  : farmfuncs.cpp- l\_farm\_cattle\_veg\_reduce2()
    : farmfuncs.cpp- l\_farm\_event\_do\_it\_prob()
      : farmfuncs.cpp- l\_farm\_herbicide\_kills()
        : farmfuncs.cpp- l\_farm\_insecticide\_kills()
          : farmfuncs.cpp- l\_farm\_pig\_veg\_reduce()
            : farmfuncs.cpp- l\_map\_rotation\_files\_prefix()
              : croprotation.cpp- l\_pest\_enable\_pesticide\_engine
                : Vole\_all.cpp- l\_pest\_insecticide\_amount
                  : farmfuncs.cpp- l\_pest\_product\_0\_amount
                    : farmfuncs.cpp- l\_pest\_product\_1\_amount
                      : farmfuncs.cpp- l\_pest\_productapplic\_period
                        : farmfuncs.cpp- l\_pest\_productapplic\_startdate
                          : farmfuncs.cpp- last\_treatment
                            : treatment.h

---

Generated on Thu Jan 22 14:13:48 2009 for ALMaSS ODDox by 
 1.5.6
